# CuO-based materials for thermochemical redox cycles: the influence of the formation of a CuO percolation network on oxygen release and oxidation kinetics

**DOI:** 10.1007/s43938-022-00013-2

**Published:** 2022-10-26

**Authors:** Qasim Imtiaz, Andac Armutlulu, Felix Donat, Christoph Müller

**Affiliations:** 1 Laboratory of Energy Science and Engineering, Department of Mechanical and Process Engineering, ETH Zürich, 8092 Zurich, Switzerland; 2 Present Address: Department of Chemistry and Chemical Engineering, Lahore University of Management Sciences, Lahore, 54792 Pakistan

## Abstract

Thermochemical redox cycles such as chemical looping combustion (CLC) are an economically promising CO_2_ capture technology that rely on the combustion of a hydrocarbon fuel with lattice oxygen that is derived from a solid oxygen carrier. The oxygen carrier is typically regenerated with air. To increase the agglomeration resistance and redox stability of the oxygen carriers, the active phase is often stabilized with high Tammann temperature ceramics, resulting in the formation of so-called cermet structures. It has been hypothesized that the redox performance of the cermets depends critically on the conduction pathways for solid-state ionic diffusion and the activation energy for charge transport. Here, we investigate the influence of the formation of a percolation network on the electrical conductivity and the rate of oxidation for CeO_2_-stabilized Cu. We found that for oxygen carriers that contained 60 wt. % CuO, the charge transport occurred predominately via Cu/CuO conduction pathways. Below the percolation threshold of CuO, the conduction of charge carriers took place via CeO_2_ grains, which formed a continuous network. The measurements of charge transport and redox characteristics confirmed that the activation energy for charge transport through the cermet increased with decreasing Cu content. This indicates that the solid-state diffusion of charge carriers plays an important role during re-oxidation.

**Supplementary Information:**

The online version contains supplementary material available at 10.1007/s43938-022-00013-2.

## Introduction

Chemical looping combustion (CLC) is a promising carbon dioxide capture and sequestration (CCS) technology that may reduce CO_2_ capture costs significantly [1, 2]. For CLC, the estimated cost of CO_2_ capture is in the range 7 – 14 $ per ton of CO_2_ avoided [3]. On the other hand, the costs of CO_2_ capture using amine scrubbing, an industrially proven post-combustion CO_2_ capture technology, are estimated to be ~ 55$ per ton of CO_2_ avoided [4]. In CLC, a hydrocarbon fuel is combusted via a mechanism that shows some similarity to a Mars-van-Krevelen mechanism [5], viz*.* a hydrocarbon is combusted with the lattice oxygen of a metal oxide:

1 $${\text{C}}_{{\text{n}}} {\text{H}}_{{{\text{2m}}}} \left( {\text{g}} \right) \, + \, \left( {{\text{2n }} + {\text{ m}}} \right){\text{Me}}_{{\text{x}}} {\text{O}}_{{\text{y}}} \left( {\text{s}} \right) \, \to {\text{ nCO}}_{{2}} \left( {\text{g}} \right) \, + {\text{ mH}}_{{2}} {\text{O }}\left( {\text{g}} \right) \, + \, \left( {{\text{2n}} + {\text{m}}} \right){\text{Me}}_{{\text{x}}} {\text{O}}_{{{\text{y}} - {1}}} \left( {\text{s}} \right).$$

In the regeneration step, the reduced metal oxide is regenerated with oxygen from the gas phase:

2 $${\text{2M}}_{{\text{x}}} {\text{O}}_{{{\text{y}} - {1}}} \left( {\text{s}} \right) + {\text{O}}_{{2}} \left( {\text{g}} \right) \to {\text{2M}}_{{\text{x}}} {\text{O}}_{{\text{y}}} \left( {\text{s}} \right).$$

The regeneration reaction is performed typically using air and is exothermic. In CLC, the depletion and subsequent replenishment of lattice oxygen of a so-called oxygen carrier is split into two spatially or temporally separated half-steps. As a consequence, in CLC, after the condensation of steam, a pure stream of CO_2_ is produced inherently, thereby, reducing appreciably the costs associated with the separation of CO_2_ from a flue gas. Furthermore, an exergy analysis of the CLC process shows its superiority over the conventional combustion process [6].

However, the CLC process depends critically on the oxygen carrier’s ability to transfer oxygen via the two steps outlined above from air to the hydrocarbon fuel over multiple cycles. Therefore, the development of oxygen carriers that possess a high and stable oxygen transfer capacity is one of the main challenges in making CLC an attractive option for practical implementations. The oxygen carriers typically used for CLC include the oxides of Ni, Fe, Cu and Mn [7, 8]. In addition naturally occurring materials (such as ilmenite FeTiO_3_, Fe ores, Mn ores, Cu ores, etc*.*), perovskite-type oxides (e.g. CaMn_0.875_Ti_0.125_O_3-δ_ and CaMn_0.9_Mg_0.1_O_3-δ_) and mixed oxides (such as Mg_2_MnO_4_, Mn_7_SiO_12_, (Mn_x_Fe_1-x_)_2_O_3_, etc*.*) have been studied [9–11]. Most unsupported oxygen carriers show a rapidly decreasing redox activity with cycle number. One approach to stabilize the redox activity of the oxygen carriers is the use of cermets, i.e. metal-ceramic composites that stabilize the oxygen carrier via the incorporation of a high Tammann temperature ceramic, e.g. Al_2_O_3_, MgAl_2_O_4_, CeO_2_, etc.[12–14] The improved redox performance of supported oxygen carriers is generally attributed to the improved sintering resistance and enhanced intra-particle gaseous diffusivity [15]. However, it has also been hypothesized by Liu et al*.*[16] that the addition of an oxygen anion conducting support such as ZrO_2_ enhances the solid-state diffusion of oxygen anions and electrons within the oxygen carrier particles, which in turn assists the rate of oxidation of the reduced metal oxide.

Using DFT calculations, Li et al*.*[17, 18] argued that the rate of the reduction and oxidation reactions of the oxygen carriers also depends on the activation energy for solid-state diffusion. Although, this is true only if the rates of redox reactions are largely controlled by ionic and electronic diffusion, nonetheless, so far this aspect has received very little attention and it largely lacks experimental confirmation. To transport oxygen anions and electrons through a composite during redox reactions, a three-dimensional continuous network of electrically conductive pathways is required. Such a network is generally referred to as a percolation network [19]. At the percolation threshold a sharp drop in the electrical resistance of the material is observed owing to the formation of charge conducting bridges [19, 20]. Therefore, one would expect that the degree of percolation will affect critically the transport of electrons and oxygen anions, which in turn would influence the rate of the redox reactions. Recently, Jovanovic and Marek [21] used percolation theory to model the reduction reaction of unsupported Fe_2_O_3_ during CLC and found that solid-state diffusion plays an important role in the reduction of non-porous F_2_O_3_ particles. To our knowledge the influence of the formation of a percolation network on the rate of reduction and oxidation of an oxygen carrier has not been experimentally investigated so far.

Hence, this work is concerned with the electrical conductivity and the rate of oxidation of an oxygen carrier as a function of the percolation “degree”. To this end, we have performed experiments on model cermets that contain CuO as the active phase and CeO_2_ as the support. Choosing CuO as the active phase was motivated by the fact that CuO has a high oxygen carrying capacity of 0.20 g O_2_/g CuO, exothermic reduction reactions, a low tendency for carbon deposition and a high electrical conductivity [22]. In addition, CeO_2_ is a mixed ionic-electronic conductor (MIEC) that can also contribute to the storage and release of oxygen via the Ce^4+^–Ce^3+^ transition [23–25]. The CuO content in the materials varied in the range 20–60 wt. % CuO. The degree of percolation of CuO in the cermet structures was visualized using focused ion beam milling combined with scanning electron microscopy (FIB-SEM). To probe the conductivity of the cermets 4-point direct current (DC) conductivity measurements were acquired. The oxidation kinetics of the cermets were determined in a thermo-gravimetric analyzer at 700 °C. Combining the results of these experiments allowed us to demonstrate that the release and uptake of oxygen of the oxygen carriers depended critically on the degree of percolation of CuO in the cermet structure.

## Experimental

### Oxygen carrier synthesis

A modification of the co-precipitation technique originally reported by He et al*.* [26] was used to synthesize CeO_2_-stabilized CuO containing 20, 30 or 60 wt. % CuO. In a typical synthesis, first appropriate amounts of Cu(NO_3_)_2_∙2.5H_2_O and Ce(NO_3_)_3_∙6H_2_O were dissolved in 400 mL of deionized water (15 MΩ.cm). Subsequently, 400 mL of an aqueous solution containing 24 g NaOH and 6 g Na_2_CO_3_ was added drop wise to the nitrate solution under continuous stirring. After adjusting the pH of the resulting slurry to 8.5 using NaOH, the mixture was heated to 80 °C and kept at 80 °C for 15 h under reflux. The resulting precipitate was filtered and washed with deionized water until the electrical conductivity of the filtrate was < 100 μS/cm. The cake of the washed precipitate was dried in an oven at 100 °C for 24 h and subsequently calcined in a muffle furnace at 1000 °C for 2 h (temperature ramp of 5 °C /min). The calcined materials were crushed and sieved into two different size ranges, viz*.* 300–425 µm and 106–150 µm. Throughout this paper, the abbreviation CuxCe (x indicates the wt. % of CuO in the material) will be used to refer to the different materials synthesized.

## Characterization of the oxygen carriers

A Bruker D8 Advance X-ray diffractometer was used to determine the crystalline phases present in the freshly calcined and reduced materials. The diffractometer was mounted with a Lynx eye super speed detector and operated at 40 mA and 40 kV using CuK_α_ radiation (λ = 1.5418 nm). Each sample was scanned within the range of 2θ = 30°–70° using a step size of 0.0275º per second. The average crystallite sizes of CuO and CeO_2_ were estimated using the Scherrer equation [27]. The surface area and pore volume of the synthesized materials were calculated using, respectively, the Brunauer et al*.* [28] and Barrett et al. [29] models. A Quantachrome NOVA 4000e analyzer was used to measure the N_2_ adsorption and desorption isotherms of the synthesized materials at −196 °C. Each sample was degassed for 3 h at 300 °C prior to the measurement. The surface morphology of the freshly calcined oxygen carriers was characterized using a scanning electron microscope (Zeiss Gemini 1530 FEG) operated at 20 kV. The elemental composition of the surface was mapped using energy dispersive X-ray (EDX) spectroscopy. The degree of percolation of CuO in the synthesized materials was investigated using focused ion beam milling combined with scanning electron microscopy (FIB-SEM). Prior to FIB-SEM analysis, the samples were embedded in an epoxy resin (Epon) and cut into conical shapes. Subsequently, the specimens were affixed to an SEM stub with an electrically conductive silver paste and sputter-coated (Bal-Tec SCD 050) with Au for 1 min. During the analysis (Zeiss FIB-SEM NVision 40), the samples were tilted at an angle of 54° and the area of interest was milled with Ga ions at 30 kV and 1.5 nA. An automated serial sectioning procedure with integrated drift correction was utilized during milling and image acquisition. The acquired SEM images were tilt-corrected. N_2_-temperature programmed reduction (TPR) experiments were performed in a Mettler Toledo TGA/DSC 1 thermo-gravimetric analyzer (TGA). In a typical experiment, ~ 15 mg of the material was heated from room temperature to 1000 °C at a rate of 10 °C/min under a flow of 100 mL/min of N_2_ and kept at 1000 °C for 30 min. In all experiments, a constant N_2_ flow of 25 mL/min was used as purge flow over the micro-balance.

4-point DC conductivity measurements were used to measure the electrical conductivity, i.e. the combined electronic and ionic conductivity of the synthesized materials. First, the materials were crushed and pelletized by uni-axial (40 kN for 2 min) and isostatic (1000 kN for 2 min) pressing. The pellets were calcined at 1000 °C for 24 h using a heating and cooling rate of 2 °C/ min. The sintered pellets possessed a density of > 95% of the theoretical density. Platinum electrodes were painted on both sides of the pellets using platinum paste (C 3605 P, Heraeus GmbH). Subsequently, platinum wires were fixed in a 4-point electrode arrangement to the pellet using a ceramic binder [30]. The resistance of each pellet was measured in air as a function of temperature (from 25 to 950 °C using a heating and cooling rate of 3 ºC/min) by applying a DC voltage of 1 V (Keithley 2601B SMU). Three heating and cooling cycles were performed.

### Oxidation kinetics

The rate of oxidation of CeO_2_-supported Cu was measured in a TGA at 700 ºC in the kinetic regime. A mixture of 10 vol. % H_2_ in N_2_ was used for reduction, whereas re-oxidation was performed with 10.5 vol. % O_2_ in N_2_. The reaction chamber was purged with N_2_ for 30 s after each reduction and oxidation segment. The total flow rate of the gases in each reaction segment was 175 mL/min, as measured at 25 °C and 1 bar (including the 25 mL/min purge flow, N_2_, over the microbalance). In a typical experiment, ~ 5 mg of the material was placed in an alumina crucible and heated to 700 °C in air. After stabilization of the temperature, the flow of air was switched off and the material was reduced in H_2_ for 120 s. Subsequently, the sample was re-oxidized in O_2_ for 120 s and the process was repeated 20 times to assess whether the redox characteristics varied with cycle number.

## Results

### Composition and morphology of the unreacted materials

X-ray diffraction confirms that the as-synthesized materials contained CuO and CeO_2_ (Figure S1). The average crystallite sizes of CuO and CeO_2_ were estimated from the (− 111) and (111) crystal planes of CuO and CeO_2_, respectively [31]. Table 1 summarizes the crystallite sizes, surface area and pore volume of the materials. The crystallite size of CuO was not affected by the composition of the material. On the other hand, the average crystallite size of the ceramic phase, CeO_2_, decreased with increasing CuO content by up to 25%. Owing to the high calcination temperature of 1000 °C, the synthesized materials possessed a relatively low surface area (< 1 m^2^/g) and pore volume (< 0.01 cm^3^/g).

**Table 1:** Surface area, pore volume and average crystallite sizes of CuO and CeO_2_ in the synthesized materials

Material	Cu20Ce	Cu30Ce	Cu60Ce
Surface area, m^2^/g	< 1		
Pore volume, cm^3^/g	< 0.01		
CuO _(− 111)_, nm	31	30	31
CeO_2 (111)_, nm	79	75	58

**Fig. 1:**
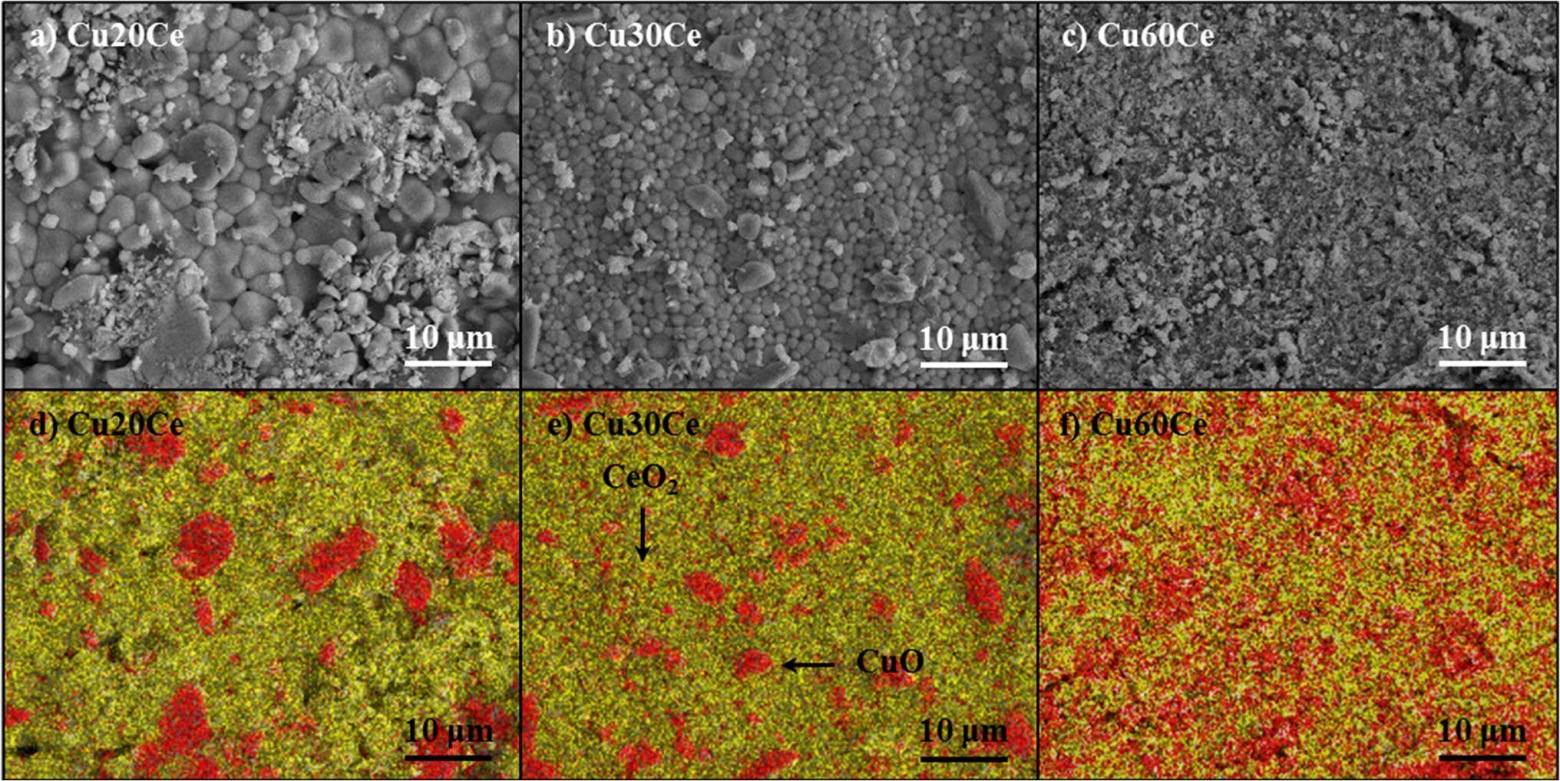
Scanning electron micrographs and the corresponding EDX maps of the oxygen carriers calcined at 1000 °C. Red and yellow colors represent CuO and CeO_2_, respectively

Scanning electron microscopy, in combination with EDX spectroscopy, was applied to analyze the surface morphology and the composition of the freshly calcined materials. Electron micrographs (Fig. 1a–c) show that the surface of the materials was composed of tightly packed micrometer-sized grains. The average size of the grains decreased with increasing quantity of CuO, viz. an average grain size of CuO of, 2.16 ± 0.77 μm, 1.82 ± 0.44 μm and 1.47 ± 0.25 μm was determined for, respectively, Cu20Ce, Cu30Ce and Cu60Ce. EDX scans show that in Cu20Ce and Cu30Ce (Fig. 1d and e, respectively) CeO_2_ grains are connected with each other. For oxygen carriers containing ≤ 30 wt. % CuO, CuO grains only decorated the CeO_2_ surface and did not form a percolation network. Increasing further the content of CuO in the oxygen carrier led to a higher degree of connectivity between the CuO and CeO_2_ grains. For example, in Cu60Ce both CuO and CeO_2_ formed percolation networks, see Fig. 1f.

**Fig. 2:**
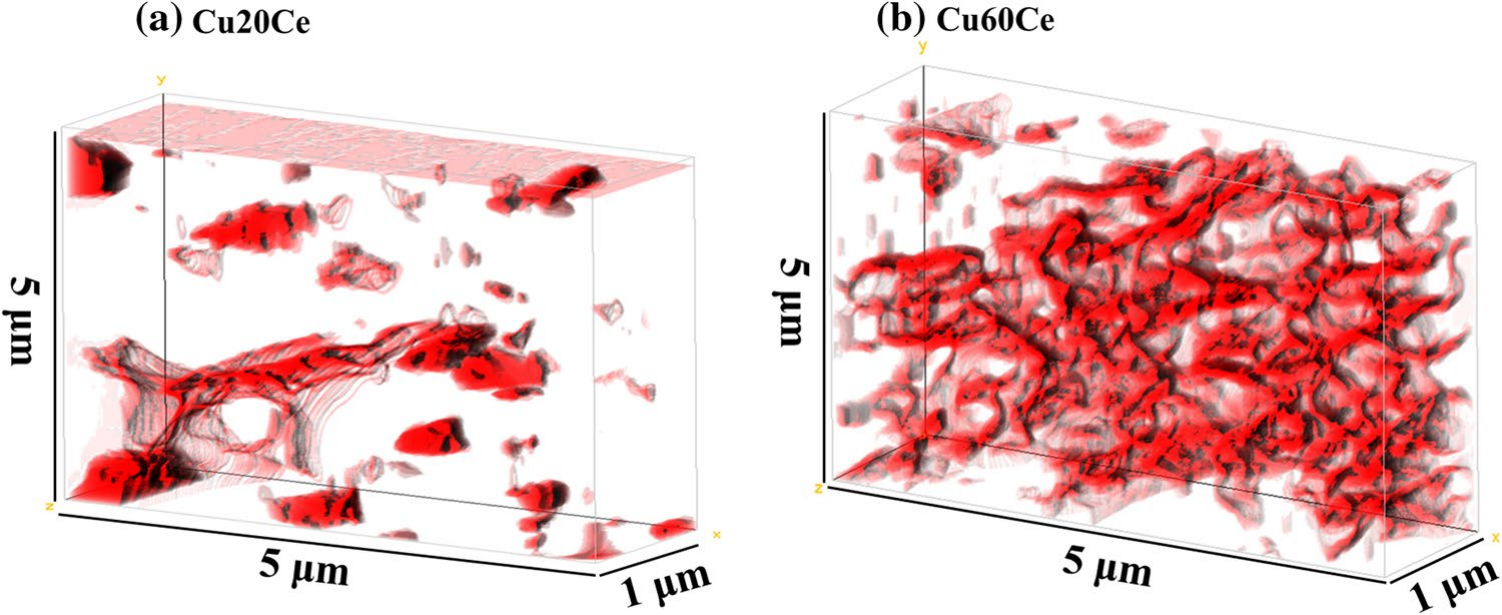
Degree of percolation of CuO in Cu20Ce and Cu60Ce obtained from FIB scanning electron microscopy

FIB-SEM was performed to visualize the degree of percolation of CuO in Cu20Ce and Cu60Ce. Three-dimensional reconstruction of the individual FIB cuts show the distribution of CuO in a volume of 25 μm^3^ of Cu20Ce and Cu60Ce (Fig. 2). In Cu20Ce (Fig. 2a) FIB-SEM reveals the presence of isolated CuO particles in a CeO_2_ matrix, indicative that the quantity of CuO in this material is below the percolation threshold to from a three-dimensional continuous network of CuO. On the other hand, Fig. 2b clearly shows that a percolation network of CuO is present in Cu60Ce, in agreement with Fig. 1f.

### Conductivity measurements

**Fig. 3:**
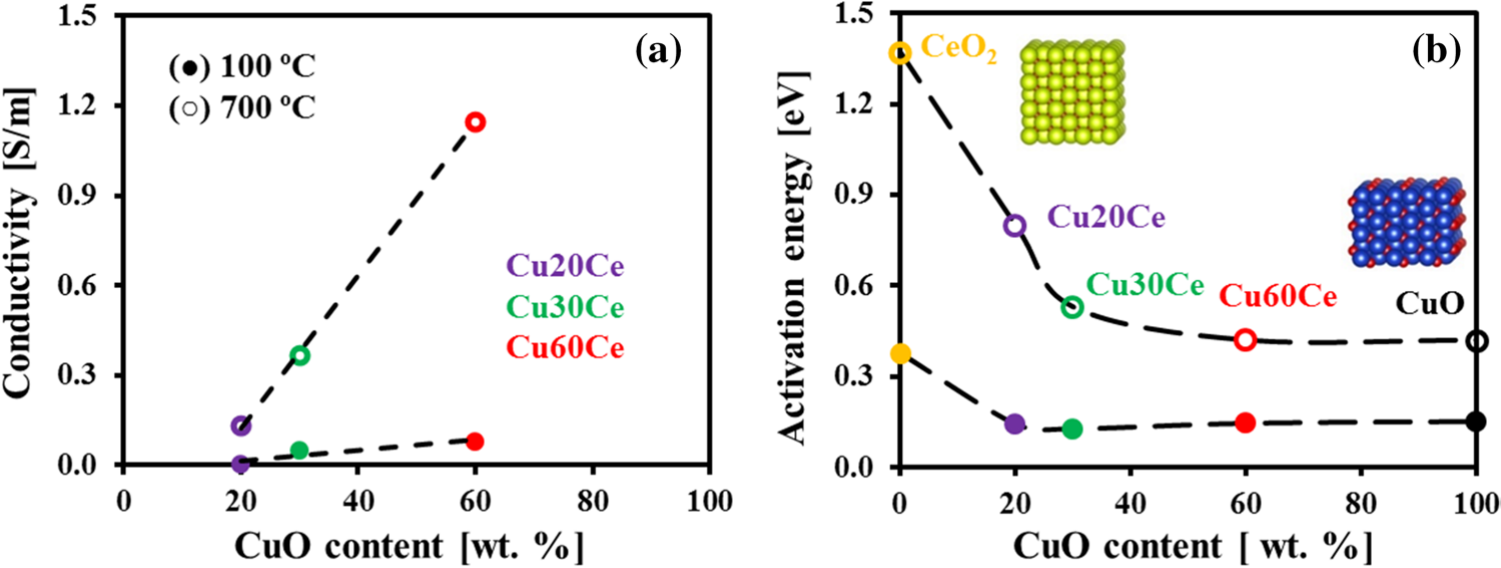
Conductivity characterization of the materials synthesized: **a** Electrical conductivity as a function of the CuO content and **b** activation energy for charge transport as a function of the CuO content in (black filled circle) low temperature (T < 450 °C for CuCe and pure CuO and T < 250 °C for pure CeO_2_) and (white filled circle) high temperature (T > 550 °C for CuCe and pure CuO and T > 300 °C for pure CeO_2_) regimes

Figure S2(a) plots the electrical conductivity (σ) of the materials as a function of temperature. For all of the materials tested the conductivity increased with increasing temperature (CuO is a p-type semiconductor) and increasing CuO content (Fig. 3a). To determine the activation energy for charge transport, the conductivity data was analyzed through an Arrhenius relationship:

3 $${\upsigma }\;{ = }\;\frac{{{\upsigma }_{{0}} }}{{\text{T}}}\exp \left( { - \frac{{{\text{E}}_{{\text{a}}} }}{{k{\text{T}}}}} \right)$$

where, σ_0_, k and E_a_ are, respectively, a proportionality constant, the Boltzmann constant and the activation energy for electrical conductivity. Plotting ln(σT) as a function of the reciprocal temperature (1/T) allowed us to identify two regimes (Figure S2(b)) with distinct activation energies. Figure 3b plots the activation energies of the pure oxides, i.e. CuO and CeO_2_, and the oxygen carriers as a function of the CuO content in the material. For temperatures < 450 °C, an activation energy in the range 0.13–0.15 eV was determined for both pure CuO and the oxygen carriers. On the other hand, in the low temperature (T < 250 °C) regime CeO_2_ had a higher activation energy for charge transport, viz*.* 0.38 eV. At higher temperatures, the number of lattice defects in the oxides increase appreciably due to thermal disorder, resulting in turn, in an increase in the activation energy for charge trasport [32]. For temperatures exceeding 550 °C, the activation energy for charge transport in pure CuO and Cu60Ce is 0.42 eV. Decreasing the CuO content from 60 to 20 wt. % resulted in an increase in the activation energy for charge transport from 0.42 to 0.80 eV. Finally, for pure CeO_2_, an activation energy for charge transport of 1.37 eV was determined for T > 300 °C.

### Temperature programmed reduction

**Fig. 4:**
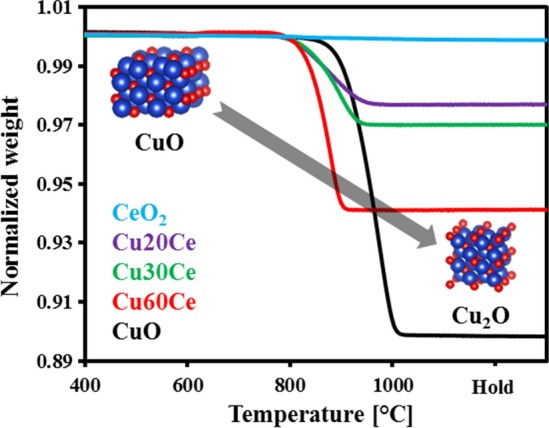
Normalized weight as a function of temperature during N_2_-TPR measurements of the materials synthesized

The oxygen release characteristics of the materials synthesized were studied by TPR experiments (N_2_ atmosphere). Figure 4 plots the normalized weight change of the materials tested as a function of temperature. For comparison, the N_2_-TPR characteristics of pure CuO and CeO_2_ are also included in Fig. 4. The reduction reaction was considered to start and finish when the material had lost 2 wt. % and 98 wt. % of the total weight loss, respectively. In the temperature range studied here, CeO_2_ was not reduced in N_2_ (in agreement with its Brouwer diagram) [33, 34]. On the other hand, the reduction of CuO (4 CuO (s) → 2 Cu_2_O (s) + O_2_ (g)) occurred in a single step in the temperature range 850–1000 °C. Turning to the oxygen carriers synthesized, the reduction of CeO_2_-stabilized CuO started at an appreciably lower temperature (~ 770 °C) when compared to that of unsupported CuO. The reduction of the oxygen carriers was also completed at a lower temperature when compared to pure CuO. For example, the reduction of Cu20Ce was completed at ~ 960 °C (~ 40 °C lower than for CuO). From Fig. 4 it can be seen that the (apparent) rate of reduction increases with increasing CuO content in the materials, viz*.* the reduction of Cu30Ce and Cu60Ce was completed at ~ 950 °C and ~ 910 °C, respectively. X-ray diffraction confirmed that only Cu_2_O and CeO_2_ were present in the reduced materials (Figure S3). Under the conditions applied here, only CuO undergoes a reduction (to Cu_2_O). Thus, the weight fraction of CuO in the materials can be determined experimentally by:

4 $${\text{CuO}}\;{\text{wt}}{.}\;{\text{fraction}}\,{ = }\,\frac{{{9}{\text{.94}}\left( {{\text{w}}_{{\text{s}}} {\text{ - w}}_{{\text{f}}} } \right)}}{{{\text{w}}_{{\text{s}}} }}$$

where w_s_ and w_f_ are the weight of the material at the start and the end of the reduction, respectively. The factor of 9.94 is derived from the stoichiometry of the reduction reaction. For all materials tested, the quantity of CuO determined by N_2_-TPR is close to the theoretically expected values (Table 2).

**Table 2:** Quantity of CuO in the oxygen carriers as determined by N_2_-TPR experiments

Material	Cu20Ce	Cu30Ce	Cu60Ce
CuO content [wt. %]	22.9	30.2	59.7

### Rate of oxidation of synthesized materials

All materials were exposed to 20 redox cycles in a TGA at 700 °C. The TGA conditions were chosen such that the rate of oxidation was not influenced appreciably by internal or external mass transfer (see SI for details). It is worth noting that the rate of oxidation of the oxygen carriers did not change significantly with cycle number (Figure S8). Figures S9(a) and 5 plot the conversion, *X*, of the oxygen carriers as a function of time and the normalized rate of oxidation as a function of time for the fifth oxidation step, respectively. The rate of oxidation was found to increase in the following order: Cu20Ce < Cu30Ce < Cu60Ce with the maximal rates of oxidation determined as ~ 0.008 mg/s/mg_Cu_, ~ 0.015 mg/s/mg_Cu_ and ~ 0.025 mg/s/mg_Cu_ for, respectively, Cu20Ce, Cu30Ce and Cu60Ce. For Cu20Ce, the rate of oxidation started to decrease for X > 0.10 (Figure S9(b)). On the other hand, the oxidation of Cu30Ce and Cu60Ce proceeded without any significant change in the rate of oxidation up to X ~ 0.55. For Cu60Ce, the rate of oxidation was constant even for X < 0.70.

**Fig. 5:**
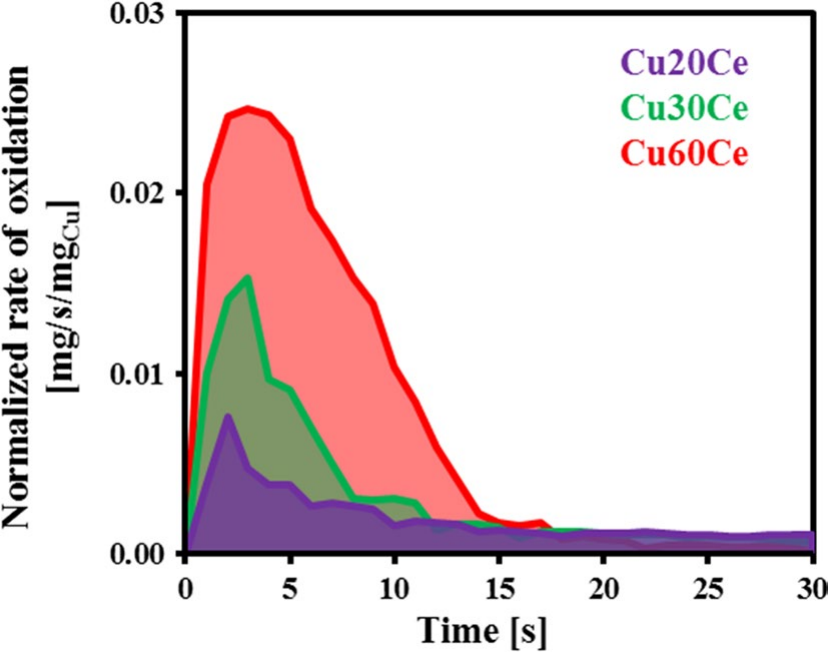
Normalized rate of oxidation of the oxygen carriers synthesized (size range 106–150 µm) as a function of time. The oxidation reaction was performed at 700 °C with 10.5 vol. % O_2_ in N_2_ (150 mL/min). The particles were placed in an alumina crucible of depth 2.9 mm and diameter 5.2 mm.

## Discussion

X-ray diffractograms of the as-synthesized (calcined) and reduced materials (Figures S1 and S3, respectively) show that CuO (or Cu_2_O) and CeO_2_ do not form solid solutions, confirming the high chemical stability of this mixture. Electrical conductivity measurements of the oxygen carriers and the references CuO and CeO_2_ reveal two distinct charge transport regimes, viz*.* a low temperature and a high temperature regime. At low temperatures (T < 450 °C) the conduction in CuO is due to the hopping of charge carriers in metal deficient CuO, i.e. Cu_1-y_O [35]. Jeong and Choi [35] determined the activation energy of charge carrier hopping in CuO as 0.1 ± 0.01 eV which is in good agreement with the values obtained here (0.13–0.15 eV). On the other hand, at low temperatures (T < 250 °C) conduction in pure CeO_2_ is due to the hopping of polarons with an activation energy of ~ 0.40 eV [36], a value that agrees very well with the value of 0.38 eV determined in this work. At high temperatures (T > 550 °C), the activation energy for conduction in unsupported CuO was determined previously as 0.7 ± 0.04 eV [35]. This value is higher than our measurements (0.42 eV) and can most likely be attributed to morphological differences between commercial CuO and the material synthesized here. The activation energy for electrical conduction in CeO_2_ at high temperatures is a function of grain size and has been determined as 0.99 eV, 1.35 eV and 2.80 eV for 10 nm, 30 nm and 5 μm sized grains [37]. The pristine CeO_2_ studied here had a grain size of ~ 100 nm and revealed an activation energy of 1.37 eV.

**Fig. 6:**
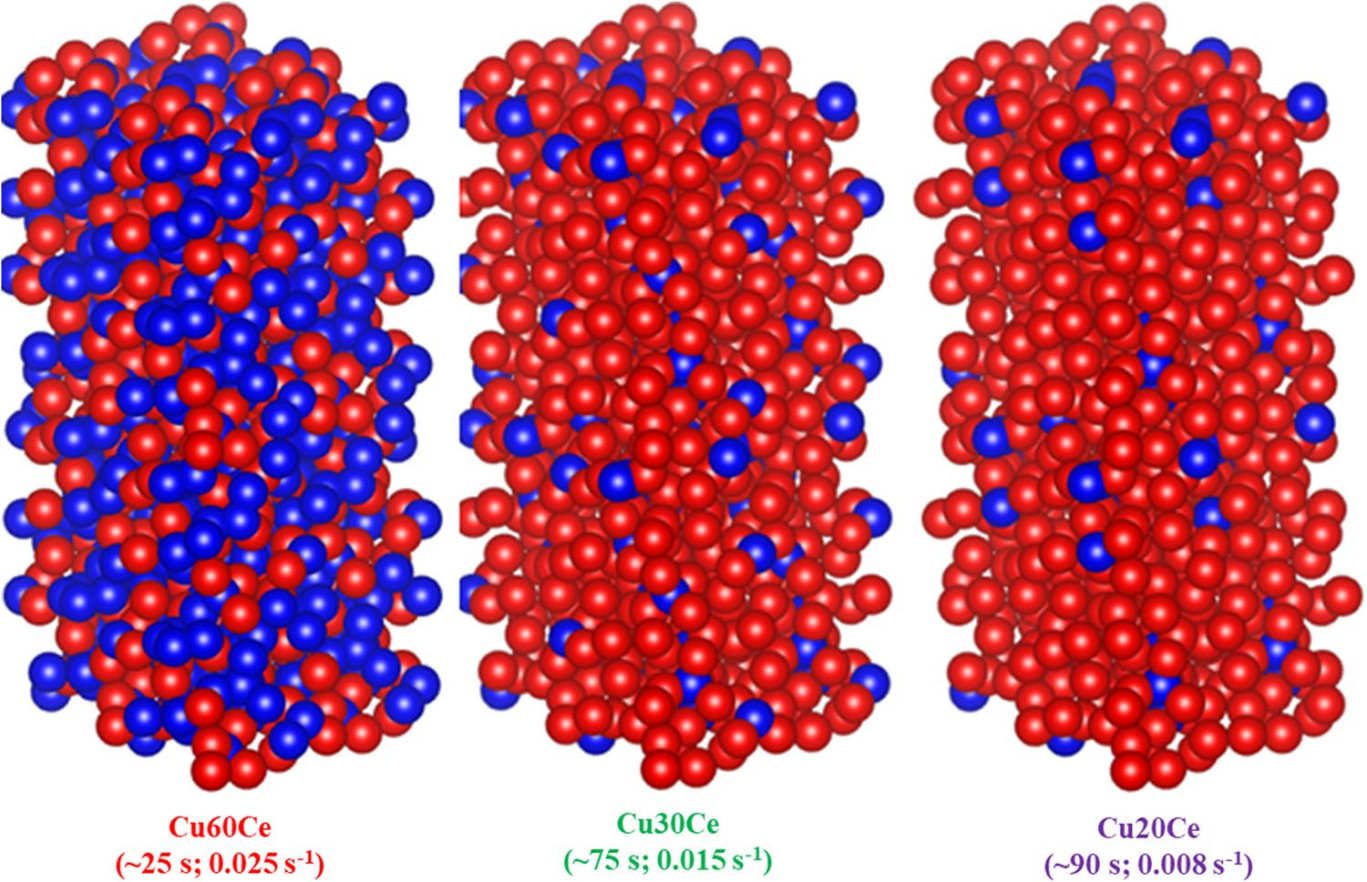
Schematic representation of the oxygen carriers synthesized. The values in parenthesis give the time required for complete oxidation and the maximal rate of oxidation. Blue and red spheres represent CuO and CeO_2_, respectively

Turning now to the charge transport measurements of the synthesized oxygen carriers, Fig. 3(b) shows that at the typical operating temperatures of the CLC process (i.e. 800–1000 °C) the activation energy for charge transport in pure CuO and Cu60Ce is identical (0.42 eV) and ~ 3 times lower than in CeO_2_. EDX mapping (Fig. 1) and FIB-SEM tomography (Fig. 2) revealed that in Cu60Ce, CuO and CeO_2_ a percolation network is formed (sketched schematically in Fig. 6). The fact that the activation energy for charge transport in Cu60Ce and pure CuO is identical, suggests that charge transport occurs through CuO conduction bridges forming a conduction pathway with a low energy barrier. When the quantity of CuO is reduced to 30 wt. % the overall conductivity of the materials decreases (Fig. 3a). However, at the same time the activation energy for charge transport increases by only 0.11 eV (Fig. 3b), indicating that charge transport in Cu30Ce occurs still predominantly through CuO conduction pathways. Although the EDX map of Cu30Ce shows that at the particle surface the CuO grains are not connected with each other, our conductivity data suggest that CuO conduction bridges still exist in Cu30Ce, albeit being less effective than in Cu60Ce. Finally, when the CuO content in the material was decreased to 20 wt. %, the activation energy for conduction increased appreciably (from 0.53 to 0.80 eV) accompanied by a substantial decrease in conductivity. The sharp decrease in conductivity indicates that the quantity of CuO in Ce20Ce is below its percolation threshold, as confirmed by EDX mapping (Fig. 1) and FIB-SEM tomography (Fig. 2), and sketched in Fig. 6. As a consequence, in Cu20Ce charge transport occurs predominately through CeO_2_ conduction pathways, yielding in turn a high activation energy for charge transport (0.80 eV). Our conductivity measurements reveal that the percolation threshold of CuO is between 20 and 30 wt. %. The minimum quantity of CuO required to form a percolation network can be estimated using percolation theory, developed to determine the conductivity threshold in a binary composite of conducting and insulating particles. According to Kryuchkov [38], the critical volume fraction of the conducting material (here CuO) depends on the ratio of the size of the conducting (i.e., CuO) and non-conducting particles (here CeO_2_) and can be calculated according to:

5 $$V_{c} = \frac{1}{{5.55 + 3\frac{{d_{i} }}{{d_{c} }}}} + 0.04$$

Here, *d*_*i*_ is the diameter of the insulating particles (i.e., CeO_2_) and *d*_*c*_ is the diameter of the conducting material (i.e., CuO). The size of pure CuO and CeO_2_ particles was ~ 1.24 μm and ~ 0.11 μm, respectively, leading to a threshold value of 21.1 vol. % equivalent to 18.2 wt. % CuO. This value is at the lower side of our experimentally determined percolation threshold in the range 20–30 wt. % CuO. The difference between the experimentally and theoretically estimated percolation threshold can be explained by the difference in the particles size of pure CeO_2_ (~ 0.11 μm) and CeO_2_ present in the oxygen carriers (average particle size ~ 1.47 μm).

From the N_2_-TPR profiles of the synthesized materials (Fig. 4) it is evident that the rate of oxygen release increased with increasing CuO content in the material. However, the TPR and conductivity experiments were performed in N_2_ and air, respectively. Therefore, it is not possible to establish a relationship between the rate of oxygen release during N_2_-TPR and the activation energy for charge transport. Thus, in this work attempts were made to relate the rate of oxidation to the conductivity performance of the materials. Our results (Figures S8 and 5) show that the normalized rates of oxidation decrease in the following order: Cu60Ce > Cu30Ce > Cu20Ce. The oxidation of the oxygen carriers can be divided into two regions, viz*.* surface oxidation and bulk oxidation, in accordance with the findings of Chuang et al. [39] which showed that the oxidation of Al_2_O_3_-stabilized Cu follows a shrinking core model. Since all materials possessed a low surface area (< 1 m^2^/g), it is likely that the surface oxidation occurs at identical conditions for all cermets, leading in turn to very similar rates of (surface) oxidation. Once an initial CuO layer is formed around the particle, the oxidation of the unreacted bulk Cu takes place either by the diffusion of gaseous oxygen through the CuO layer or by the transport of unreacted Cu from the bulk to the surface via CuO and/or CeO_2_ conduction bridges. It is worth mentioning here that all materials have a low pore volume (< 0.01 cm^3^/g), see Table 1, and that the rate of oxidation was not limited by intra-particle mass transfer (Figure S4). Therefore, we can assume that the oxidation of bulk Cu was not controlled by the diffusion of gaseous oxygen through CuO. Indeed, using DFT calculations and inert marker experiments, Li et al*.* [18] have demonstrated numerically that solid-state electronic and ionic conduction influences the rate of the redox reactions to a larger extent than intra-particle diffusion of the reactive (or product) gas. As outlined above, CuO-based conduction pathways have a lower energy barrier for charge transport when compared to CeO_2_-based conduction pathways. Therefore, the energy barrier for solid-state conduction is lowest for Cu60Ce due to the formation of a percolation network, yielding in turn the highest rate of oxidation. Decreasing the quantity of CuO in the material reduces the connectivity of CuO-CuO bridges, leading to an increase in the energy barrier for the transport of charge carriers. Due to the increasing activation energy for charge transport, the rate of bulk oxidation decreases with a decreasing quantity of CuO. Based on these observations, we can conclude that for the oxidation of CeO_2_-stabilized CuO (containing up to 60 wt. % CuO) the counter-diffusion of oxygen ions and electrons from the surface to the bulk are the rate-determining steps. It is worth mentioning here that the results of this study cannot be extrapolated to the unsupported CuO system as both CuO and Cu have very low Tammann temperatures of 526 °C and 405 °C, respectively, and, hence, sinter already during the first CLC cycle [9]. As a result, the morphology of the unsupported CuO and Cu under reaction conditions is very different to that of supported Cu. Secondly, the electrical properties of unsupported metals/metal oxides are very different to that of supported metals and metal oxides. For example, the rate of inward diffusion of oxygen in pure Cu and pure CuO is negligible compared to the outward diffusion of Cu/Cu^2+^ through p-type metal deficit CuO [40, 41]. Therefore, we speculate that for pure Cu the rate of outward diffusion of copper species from the bulk to the surface is significantly lower than that of supported Cu.

## Conclusion

In this work, we probed the effect of the formation of a CuO percolation network on the conduction properties and the oxidation kinetics of CeO_2_-stabilized CuO. Using a combination of EDX spectroscopy and electrical conductivity measurements we could demonstrate that the percolation threshold of CuO is between 20 and 30 wt. % and the activation energy for electrical conduction decreases with increasing CuO content due to a shift in the active conduction pathway. Above the percolation threshold of CuO, conduction takes place via CuO grains that form a continuous network. On the contrary, below the percolation threshold of CuO, solid-state diffusion occurs via CeO_2_ bridges that have a higher energy barrier for charge transport when compared to CuO bridges. Redox experiments showed that the normalized rate of oxidation increased with increasing CuO content due to a decrease in the activation energy for solid-state diffusion, indicating that the transport of charge carriers plays an important role during re-oxidation.

## Supplementary Information

Below is the link to the electronic supplementary material.

Supplementary file1 (DOCX 334 KB)

## Data Availability

The datasets generated during and/or analyzed during the current study are available from the corresponding author on reasonable request.

## References

[1] Hossain MM, de Lasa HI (2008). Chemical-looping combustion (CLC) for inherent CO_2_ separations—a review. Chem Eng Sci.

[2] Zhu X (2020). Chemical looping beyond combustion—a perspective. Energy Environ Sci.

[3] Ekstrom C, et al. Techno-economic evaluations and benchmarking of pre-combustion CO(2) capture and oxy-fuel processes developed in the European ENCAP project. Greenhouse Gas Control Technologies 9. 2009;1(1):4233–40.

[4] Tuinier MJ, Hamers HP, Annaland MV (2011). Techno-economic evaluation of cryogenic CO_2_ capture—a comparison with absorption and membrane technology. Int J Greenhouse Gas Control.

[5] Doornkamp C, Ponec V (2000). The universal character of the Mars and Van Krevelen mechanism. J Mol Catal A Chem.

[6] Ishida M, Jin HG (1994). A new advanced power-generation system using chemical-looping combustion. Energy.

[7] Adanez J (2012). Progress in chemical-looping combustion and reforming technologies. Prog Energy Combust Sci.

[8] Imtiaz Q, Kierzkowska AM, Muller CR (2012). Coprecipitated, copper-based, alumina-stabilized materials for carbon dioxide capture by chemical looping combustion. Chemsuschem.

[9] Imtiaz Q, Hosseini D, Muller CR (2013). Review of oxygen carriers for chemical looping with oxygen uncoupling (CLOU): thermodynamics, material development, and synthesis. Energ Technol.

[10] Leion H (2009). Use of CaMn_0.875_Ti_0.125_O_3_ as oxygen carrier in chemical-looping with oxygen uncoupling. Energy Fuels.

[11] Shulman A (2011). Chemical—looping with oxygen uncoupling using Mn/Mg-based oxygen carriers—oxygen release and reactivity with methane. Fuel.

[12] Imtiaz Q (2015). Highly efficient oxygen-storage material with intrinsic coke resistance for chemical looping combustion-based CO_2_ capture. Chemsuschem.

[13] Imtiaz Q, Broda M, Muller CR (2014). Structure-property relationship of co-precipitated Cu-rich, Al_2_O_3_- or MgAl_2_O_4_-stabilized oxygen carriers for chemical looping with oxygen uncoupling (CLOU). Appl Energy.

[14] Imtiaz Q (2021). Preventing agglomeration of CuO-based oxygen carriers for chemical looping applications. ACS Sustain Chem Eng.

[15] Li FX (2011). Ionic diffusion in the oxidation of iron-effect of support and its implications to chemical looping applications. Energy Environ Sci.

[16] Liu W, Dennis JS, Scott SA (2012). The effect of addition of ZrO2 to Fe2O3 for hydrogen production by chemical looping. Ind Eng Chem Res.

[17] Galinsky NL (2013). Iron oxide with facilitated O^2-^ transport for facile fuel oxidation and CO_2_ capture in a chemical looping scheme. Acs Sustain Chem Eng.

[18] Li FX (2011). Role of metal oxide support in redox reactions of iron oxide for chemical looping applications: experiments and density functional theory calculations. Energy Environ Sci.

[19] Galinski H (2011). Nonlinear oxidation kinetics of nickel cermets. Acta Mater.

[20] Clerc JP (1990). The electrical-conductivity of binary disordered-systems, percolation clusters, fractals and related models. Adv Phys.

[21] Jovanovic R, Marek EJ (2021). Percolation theory applied in modelling of Fe2O3 reduction during chemical looping combustion. Chem Eng J.

[22] Imtiaz Q (2012). Synthesis of Cu-rich, Al_2_O_3_-stabilized oxygen carriers using a coprecipitation technique: redox and carbon formation characteristics. Environ Sci Technol.

[23] Rupp JLM, Scherrer B, Gauckler LJ (2010). Engineering disorder in precipitation-based nano-scaled metal oxide thin films. Phys Chem Chem Phys.

[24] Rupp JLM, Gauckler LJ (2006). Microstructures and electrical conductivity of nanocrystalline ceria-based thin films. Solid State Ionics.

[25] Shi YU (2015). The effect of mechanical twisting on oxygen ionic transport in solid-state energy conversion membranes. Nat Mater.

[26] He L (2009). Co-Ni catalysts derived from hydrotalcite-like materials for hydrogen production by ethanol steam reforming. Top Catal.

[27] Patterson AL (1939). The Scherrer formula for X-ray particle size determination. Phys Rev.

[28] Brunauer S, Emmett PH, Teller E (1938). Adsorption of gases in multimolecular layers. J Am Chem Soc.

[29] Barrett EP, Joyner LG, Halenda PP (1951). The determination of pore volume and area distributions in porous substances. 1. Computations from nitrogen isotherms. J Am Chem Soc.

[30] Rupp JLM (2014). Scalable oxygen-ion transport kinetics in metal-oxide films: impact of thermally induced lattice compaction in acceptor doped ceria films. Adv Func Mater.

[31] Zolotoyabko E, Rupp JLM, Gauckler LJ (2012). Interrelationship between grain size-induced and strain-induced broadening of X-ray diffraction profiles: what we can learn about nanostructured materials?. Scripta Mater.

[32] Okeeffe M, Moore WJ (1961). Electrical conductivity of monocrystalline cuprous oxide. J Chem Phys.

[33] Ackermann S (2015). Kinetics of CO_2_ reduction over nonstoichiometric ceria. J Phys Chem C.

[34] Kuhn M (2013). Structural characterization and oxygen nonstoichiometry of ceria-zirconia (Ce_1-x_Zr_x_O_2-δ_) solid solutions. Acta Mater.

[35] Jeong YK, Choi GM (1996). Nonstoichiometry and electrical conduction of CuO. J Phys Chem Solids.

[36] Tuller HL, Nowick AS (1977). Small polaron electron-transport in reduced CeO_2_ single-crystals. J Phys Chem Solids.

[37] Kosacki I (2000). Electrical conductivity of nanocrystalline ceria and zirconia thin films. Solid State Ionics.

[38] Kryuchkov YN (2000). Percolation estimation of the conductivity and elasticity of heterogeneous two-phase systems. Theor Found Chem Eng.

[39] Chuang SY (2010). Kinetics of the oxidation of a co-precipitated mixture of Cu and Al_2_O_3_ by O_2_ for chemical-looping combustion. Energy Fuels.

[40] Park JH, Natesan K (1993). Oxidation of copper and electronic transport in copper oxides. Oxid Met.

[41] Zheng C (2020). Insight into the oxidation mechanism of a Cu-based oxygen carrier (Cu → Cu2O → CuO) in chemical looping combustion. Energy Fuels.

