# Supplementary material for: CuO-based materials for thermochemical redox cycles: the influence of the formation of a CuO percolation network on oxygen release and oxidation kinetics
Source: Discov Chem Eng. 2022 Oct 26;2(1):6. doi: 10.1007/s43938-022-00013-2 (PMC9625079; doi:10.1007/s43938-022-00013-2)
Supplement: Supplementary file 1 — Supplementary file1 (DOCX 334 KB) [file 43938_2022_13_MOESM1_ESM.docx]

**Supplementary file**

**CuO-based materials for thermochemical CO_2_ capture cycles: The influence of the formation of a CuO percolation network on oxygen release and oxidation kinetics**

Qasim Imtiaz, Andac Armutlulu, Felix Donat and Christoph R. Müller*

Laboratory of Energy Science and Engineering, Department of Mechanical and Process Engineering, ETH Zurich, Leonhardstrasse 27, 8092 Zurich, Switzerland

*****Corresponding Author

muelchri@ethz.ch

**S1. X-ray diffractograms of the as-synthesized oxygen carriers**


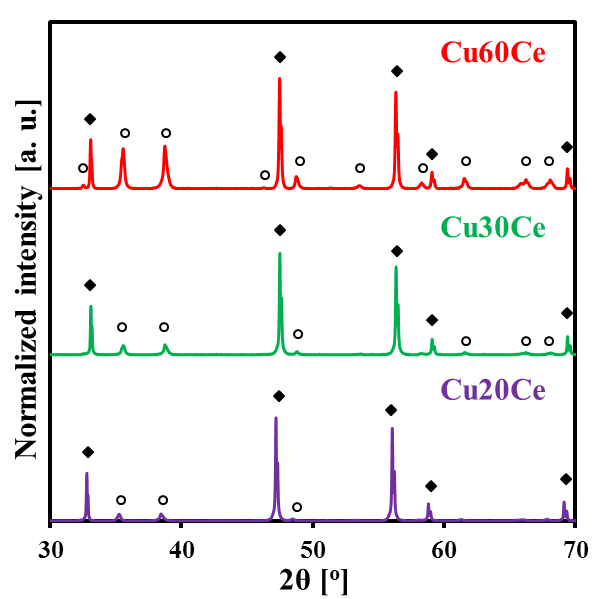


**Figure S1.** X-ray diffractograms of the as-synthesized (calcined) oxygen carriers.
The following compounds were identified: (**○**) CuO and (♦) CeO_2_. It should be noted that the shoulder in the CeO_2_ peaks is due to a K_α2_ transition and does not indicate the reduction of Ce^4+^ to Ce^3+^.

**S2. Conductivity data of the synthesized materials**


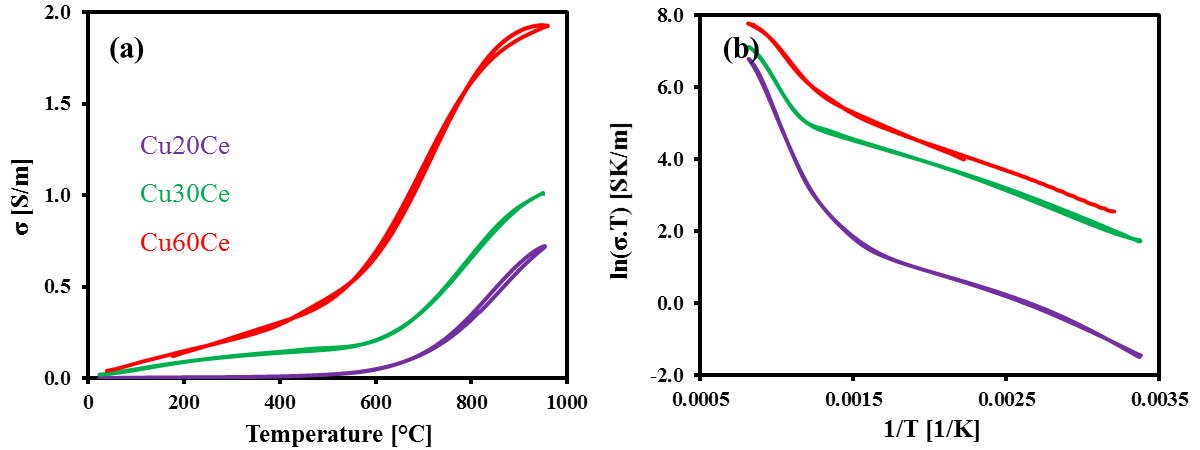


**Figure S2.** (a) Electrically conductivity of the materials synthesized as a function of temperature and (b) ln(σ.T) as a function of reciprocal temperature.

**S3. X-ray diffractograms of the reduced oxygen carriers**


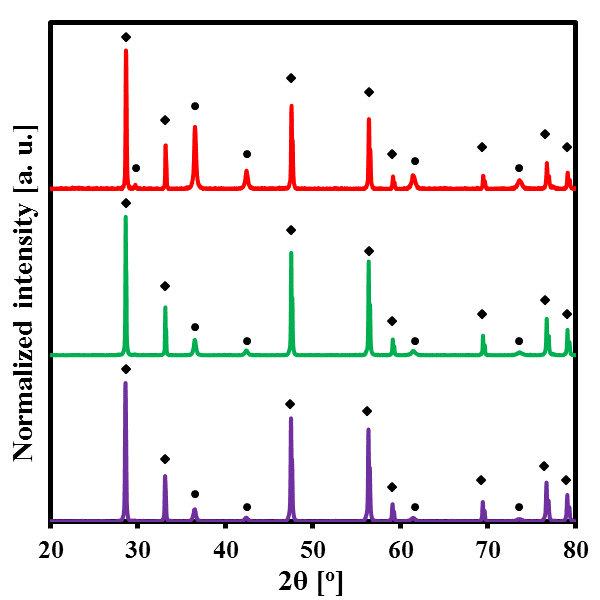


**Figure S3.** X-ray diffractograms of the reduced oxygen carriers: (**—**) Cu20Ce, (**—**) Cu30Ce and (**—**) Cu60Ce. The following compounds were identified: (●) Cu_2_O and (♦) CeO_2_. It should be noted that the shoulder in CeO_2_ peaks is due to a K_α2_ transition and does not imply the reduction of Ce^4+^ to Ce^3+^.

**S4. Effect of particle diameter on the rate of oxidation**

To assess the influence of the particle diameter on the rate of oxidation, four batches of Cu60Ce (~5 mg) with different particle sizes were oxidized with 10.5 vol. % O_2_ in N_2_ at 700 ºC in a TGA, Figure S4. We observe only a very small difference in the measured rates of oxidations for particles diameters 106 – 150 µm and 150 – 212 µm. This observation suggests that for particle sizes < 212 µm, internal mass transfer limitations are not significant. For particles in the size range 212 – 300 µm, the maximum rate dropped by ~7 % and the time required to fully oxidize the particles increased from 25 s to 33 s. This indicates that for particle diameters
> 212 µm intra-particle mass transfer limits the rate of oxidation. For particle diameters
300 – 425 µm, the time required to fully oxidize the material increased further to 87 s, accompanied by a drop of ~24 % in the maximal rate of oxidation, indicating significant internal mass transfer limitations. Based on the results shown in Figure S4, particles in the size range 106 – 150 µm were used in the subsequent experiments.


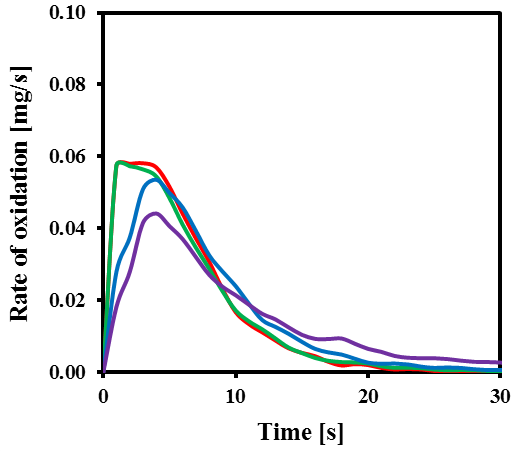


**Figure S4.** Normalized rate of oxidation of ~5 mg Cu60Ce with different particle sizes:
(**—**) 106 – 150 µm, time required for complete oxidation: 22 s; (**—**) 150 – 212 µm, time required for complete oxidation: 25 s; (**—**) 212 – 300 µm, time required for complete oxidation: 33 s; (**—**) 300 – 425 µm, time required for complete oxidation: 87 s. The oxidation reaction was performed at 700 ºC under a flowrate of 150 mL/min of 10.5 vol. % O_2_ in N_2_ in an alumina crucible (depth 2.9 mm and diameter 5.2 mm).

**S5. Effect of particle – particle interaction**

To minimize the effect of neighboring particles on the rate of oxidation only a monolayer of particles was used in all of the experiments. To confirm that the reaction of a particle was not affected by the presence of neighboring particles, two samples of Cu60Ce (106 – 150 µm,
~5 mg) were oxidized at 700 °C under identical conditions, except that the second sample was mixed with ~5 mg of alumina particles (106 – 150 µm) to increase the particle-particle spacing. The oxidation rates and the time required to fully oxidize the oxygen carrier were found to be very similar (Figure S5). Based on the results obtained we can conclude that neighboring particles do not have an appreciable influence on the oxidation characteristics.


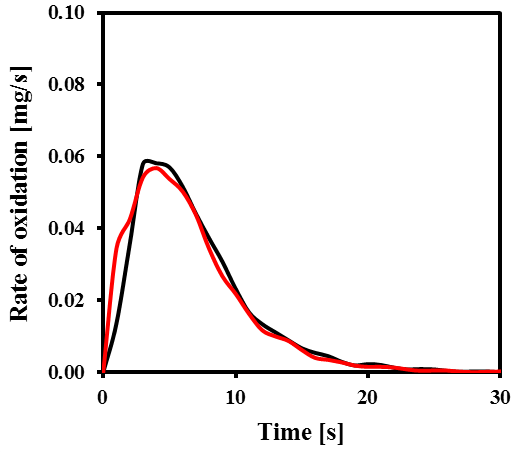


**Figure S5.** Normalized rate of oxidation of particles in the size range 106 – 150 µm:
(**—**) ~5 mg Cu60Ce, time required for complete oxidation: 25 s and (**—**) ~5 mg Cu60Ce + ~5 mg Al_2_O_3_, time required for complete oxidation: 25 s. The oxidation reaction was performed at 700 °C under a flow rate of 150 mL/min of 10.5 vol. % O_2_ in N_2_ in an alumina crucible (depth 2.9 mm and diameter 5.2 mm).

**S6. Rate of external mass transfer**


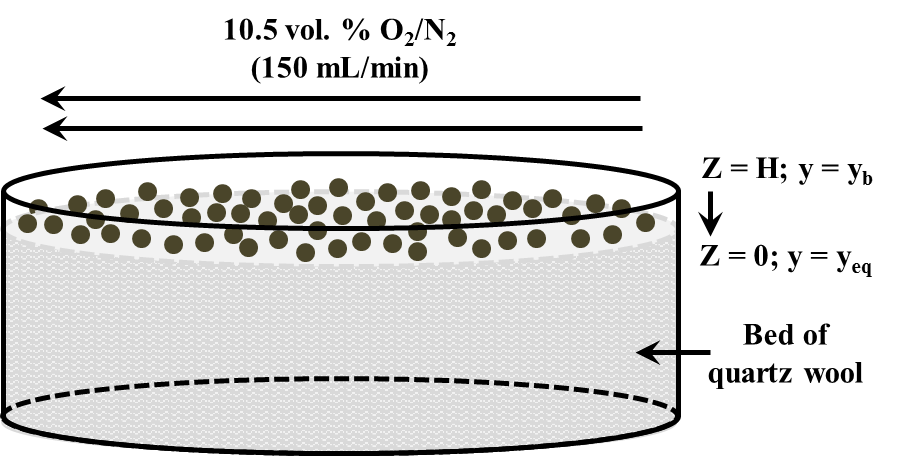


**Figure S6.** Schematic diagram of a TGA crucible containing oxygen carrier particles.

To determine whether the oxidation rates measured in a TGA are influenced by external mass transfer limitations, the rate of external mass transfer was estimated assuming that the mass transfer in the crucible is by diffusion only, *i.e.* ignoring fluid dynamic effects, such as convection. As shown in Figure S6, the arrangement of the sample particles in a TGA crucible resembles that of a Stefan tube. Therefore, the Stefan-Maxwell equation (equation 1) can be used to estimate the flux of nitrogen and oxygen between the top of the crucible and the external surface of the particles.

 (1)

Here $y_{O_{2}}$ and $y_{N_{2}}$ are, respectively, the mole fractions of oxygen and nitrogen, *c_t_* is the gas concentration in the crucible, *D* denotes the binary molecular diffusivity of oxygen in nitrogen, $N_{O_{2}}$ and $N_{N_{2}}$ are the molar fluxes of oxygen and nitrogen, respectively, and *z* is the space coordinate (pointing from the top to the bottom of the crucible). Here we assume that at the top of the crucible the mole fraction of oxygen is constant and equal to that of the bulk gas mixture, *y_b_*, passing through the TGA furnace. Furthermore, the mole fraction of oxygen at the external surface of the particles was assumed to be at thermodynamic equilibrium (*y_eq_*) of the oxidation reaction (2Cu + O_2_ ↔ 2CuO). As nitrogen is not involved in the oxidation reaction, its diffusional downward motion must be balanced by an upward bulk motion, leading to a zero nitrogen flux in the crucible.^1^ Therefore, at steady state the molar flux of oxygen from the top of the crucible to the surface of the particle is given by:

$N_{O_{2}}=\frac{Dc_{t}}{H}\ln\left(\frac{1-y_{eq}}{1-y_{b}}\right)$ (2)

Since the experiments were performed under isothermal conditions, it was assumed that the temperature gradient between the particles and the furnace was negligible. The mole fraction of oxygen in the bulk gas was calculated by

$y_{b}=\frac{0.209F_{r}}{F_{r}+0.5F_{p}}$ (3)

where *F_r_* and *F_p_* are the molar flowrates of the reactive and purge gases, respectively. The correction factor of 0.5 was introduced to account for the fact that only about half the purge gas is mixed with the reactive gas as it flows across the crucible (depth 2.9 mm and internal diameter 5.2 mm). The average distance, H, from the top of the crucible to the surface of the particles was ~0.5 mm. The Fuller-Schettler-Giddings equation was used to estimate the binary diffusion coefficient.^2^ The flux of oxygen was calculated using equation 2 (*y_eq_* is approximately zero at 700 ºC for 2Cu + O_2_ ↔ 2CuO)^3^. Using these values an oxygen mass flux of 0.31 mg/s was obtained. The maximum rate of external mass transfer was at least by a factor of seven higher than the rate of reaction, suggesting that external mass transfer did not limit our oxidation reaction.

1. Hu W, Donat F, Scott SA, Dennis JS. Kinetics of oxygen uncoupling of a copper based oxygen carrier. Appl Energ. 2016;161:92-100.

2. Fuller EN, Schettle.Pd, Giddings JC. A new method for prediction of binary gas-phase diffusion coeffecients. Ind Eng Chem. 1966;58(5):18-27.

3. Imtiaz Q, Broda M, Muller CR. Structure-property relationship of co-precipitated Cu-rich, Al_2_O_3_- or MgAl_2_O_4_-stabilized oxygen carriers for chemical looping with oxygen uncoupling (CLOU). Appl Energ. 2014;119:557-65.

**S7. Rate of oxidation of Cu60Ce**


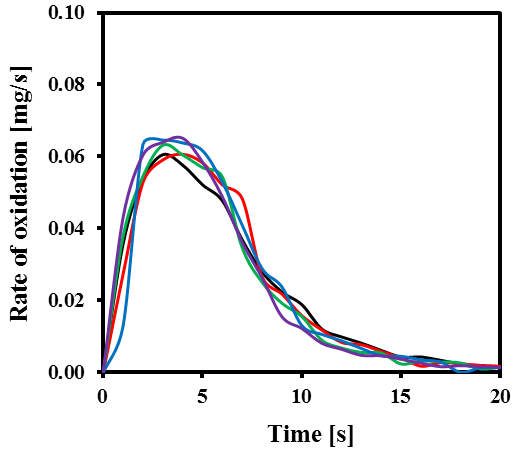


**Figure S7.** Normalized rate of oxidation of the Cu60Ce particles (size range 106 – 150 µm) as a function of cycle number: (**—**) 1^st^ cycle, (**—**) 2^nd^ cycle, (**—**) 3^rd^ cycle, (**—**) 4^th^ cycle, and
(**—**) 5^th^ cycle. The oxidation reaction was performed at 700 °C under a flowrate of
150 mL/min of 10.5 vol. % O_2_ in N_2_ in an alumina crucible with depth 2.9 mm and diameter 5.2 mm.

**S8. Oxidation kinetics of the oxygen carriers synthesized**

**
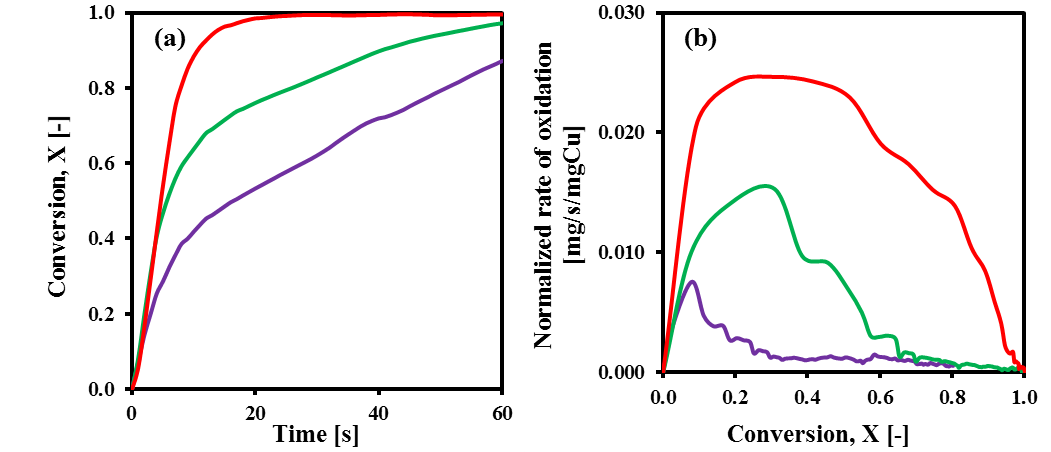
**

**Figure S8.** (a) Conversion of the oxygen carriers synthesized (size range 106 – 150 µm) as a function of time and (b) normalized rate of oxidation as a function of conversion. The oxidation reaction was performed at 700 ºC with 10.5 vol. % O_2_ in N_2_ (150 mL/min). The particles were placed in an alumina crucible of depth 2.9 mm and diameter 5.2 mm.
